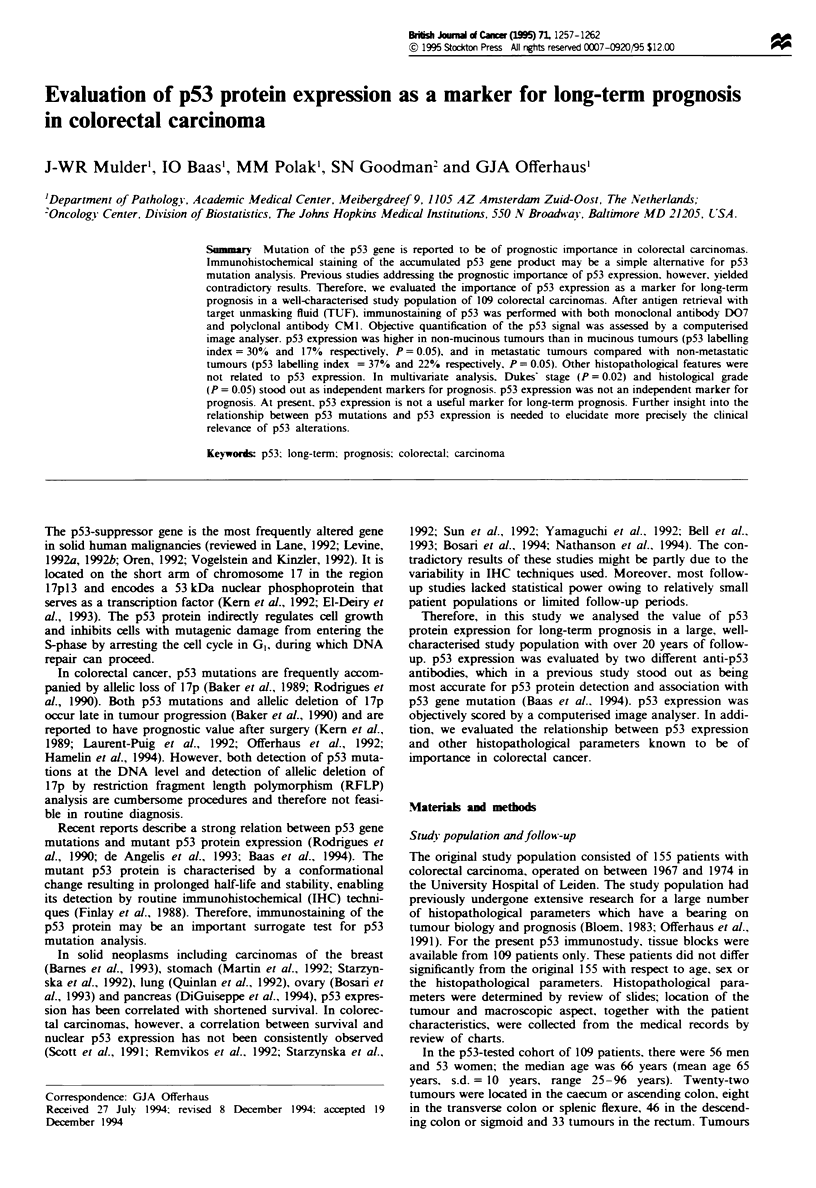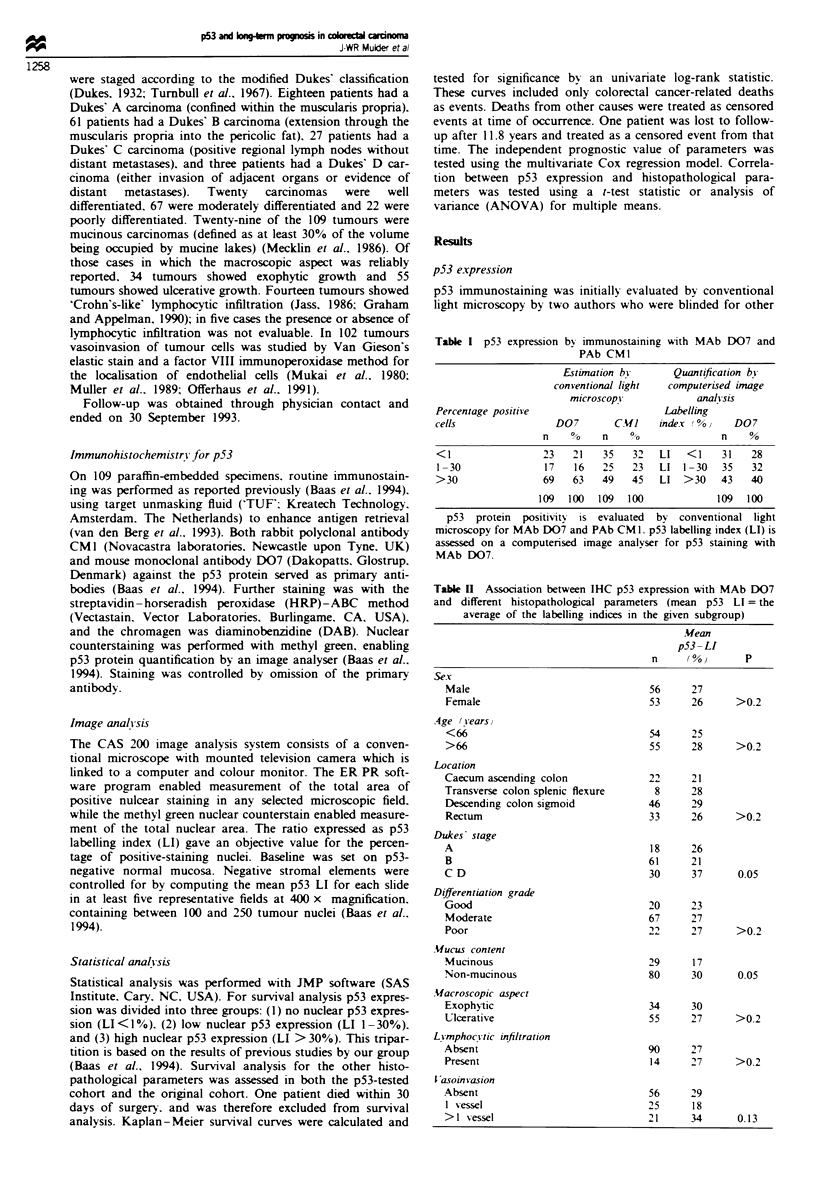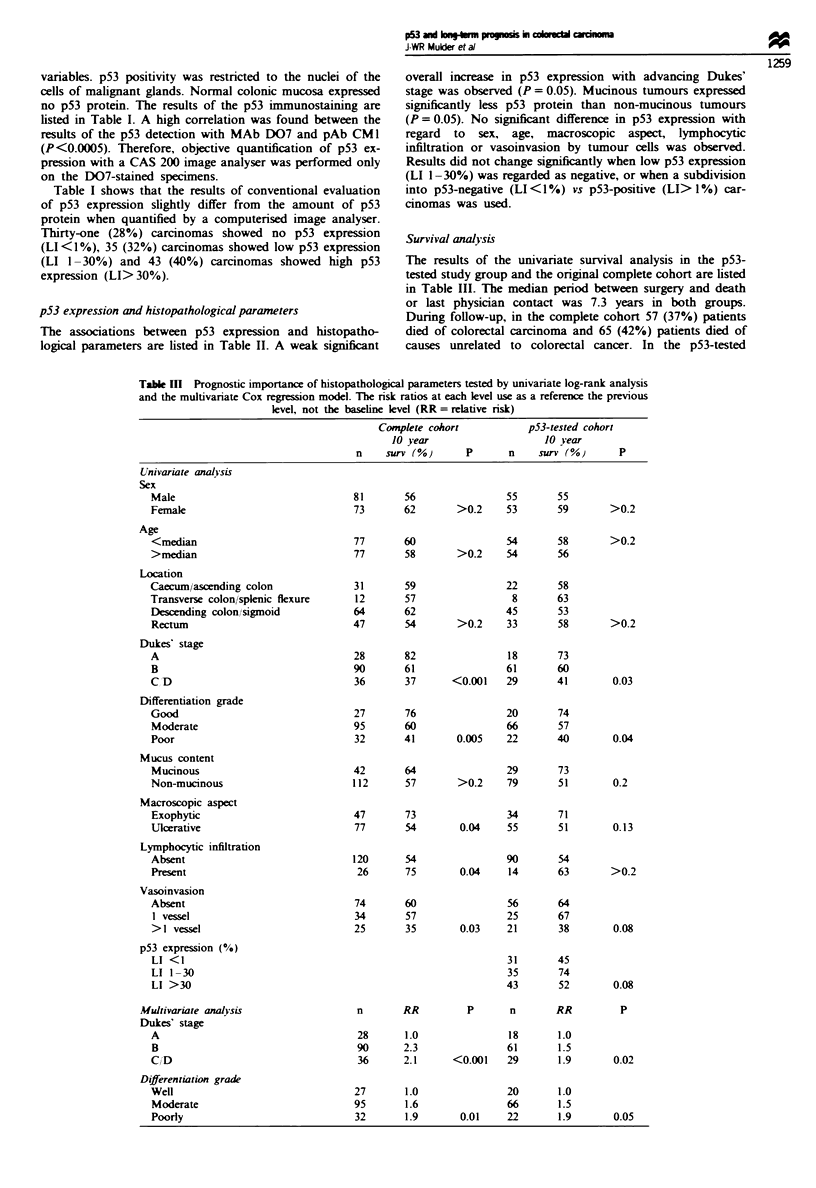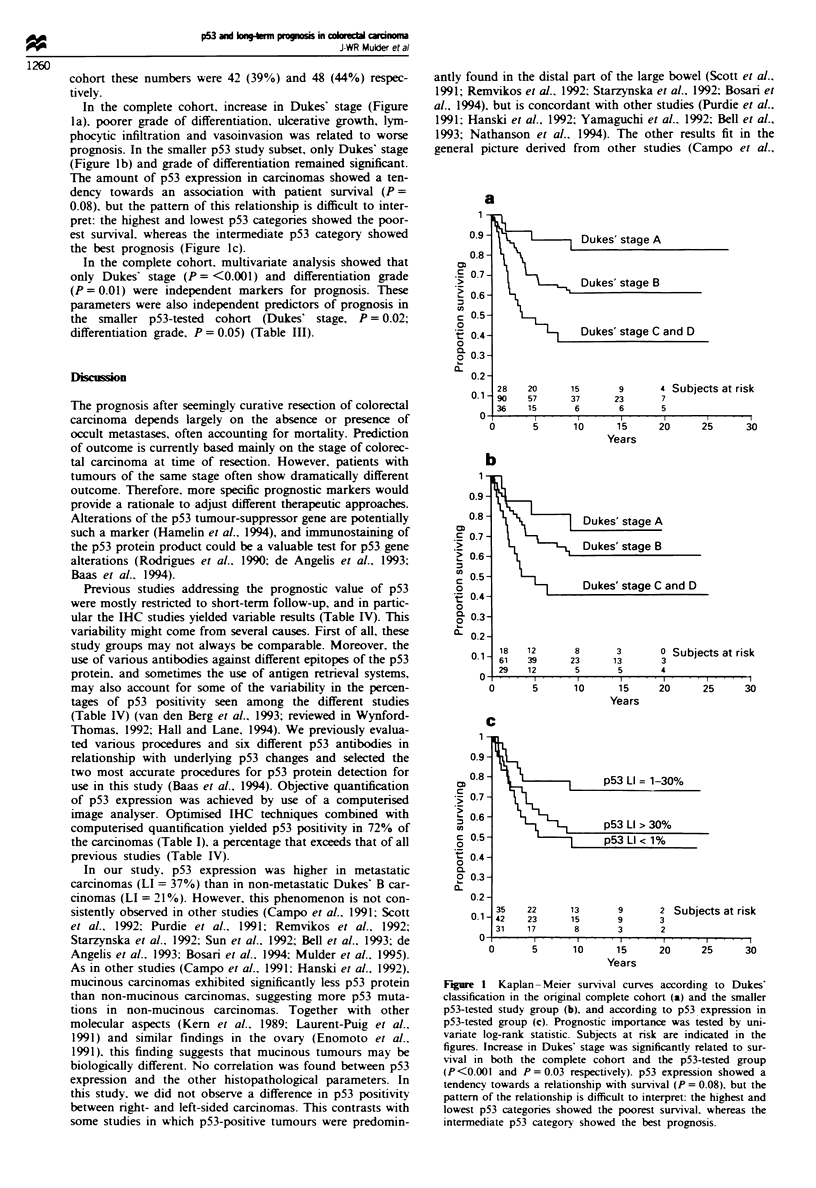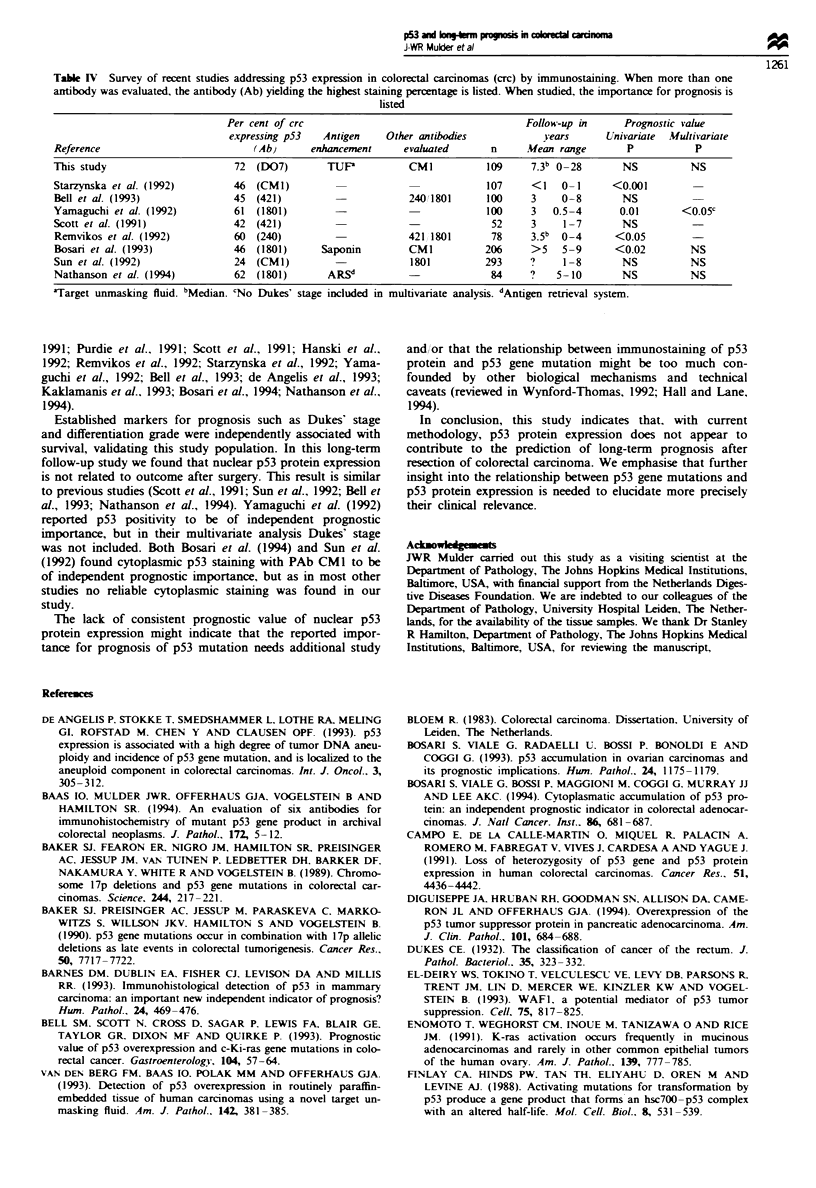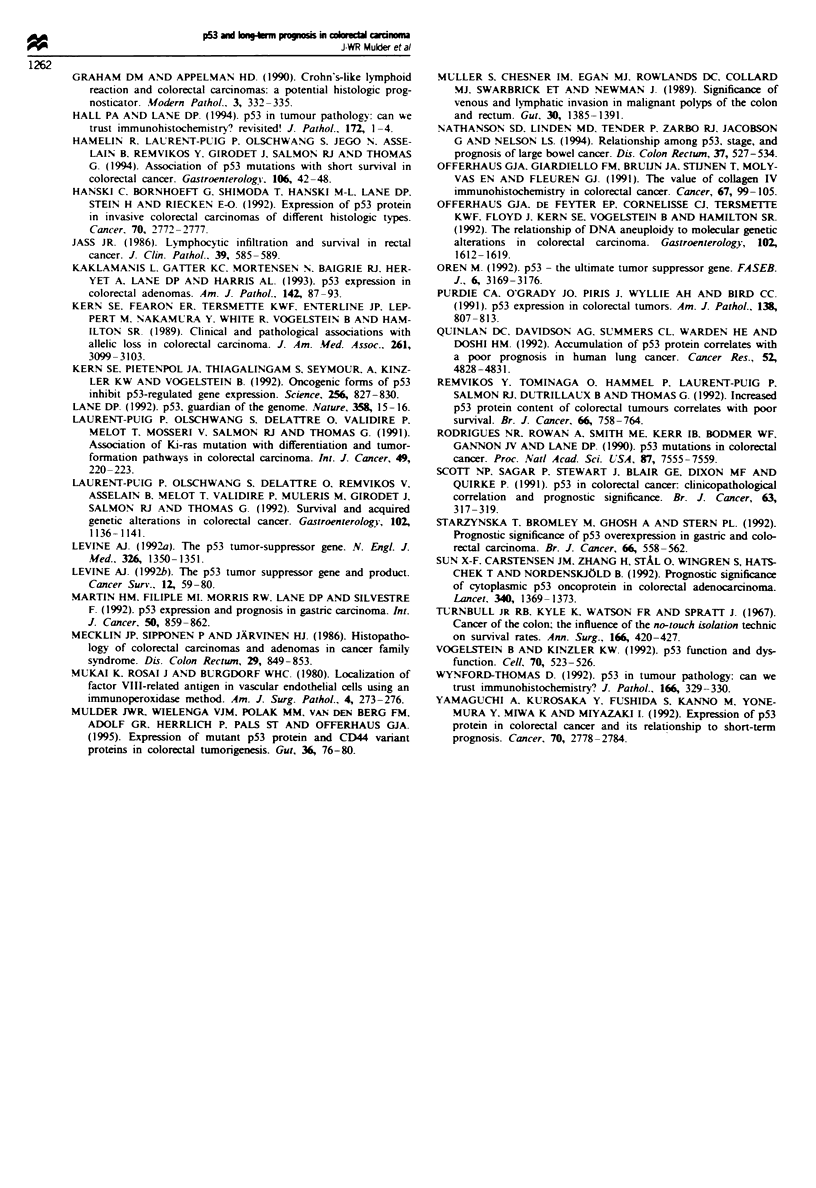# Evaluation of p53 protein expression as a marker for long-term prognosis in colorectal carcinoma.

**DOI:** 10.1038/bjc.1995.243

**Published:** 1995-06

**Authors:** J. W. Mulder, I. O. Baas, M. M. Polak, S. N. Goodman, G. J. Offerhaus

**Affiliations:** Department of Pathology, Academic Medical Center, Amsterdam Zuid-Oost, The Netherlands.

## Abstract

Mutation of the p53 gene is reported to be of prognostic importance in colorectal carcinomas. Immunohistochemical staining of the accumulated p53 gene product may be a simple alternative for p53 mutation analysis. Previous studies addressing the prognostic importance of p53 expression, however, yielded contradictory results. Therefore, we evaluated the importance of p53 expression as a marker for long-term prognosis in a well-characterised study population of 109 colorectal carcinomas. After antigen retrieval with target unmasking fluid (TUF), immunostaining of p53 was performed with both monoclonal antibody DO7 and polyclonal antibody CM1. Objective quantification of the p53 signal was assessed by a computerised image analyser. p53 expression was higher in non-mucinous tumours than in mucinous tumours (p53 labelling index = 30% and 17% respectively, P = 0.05), and in metastatic tumours compared with non-metastatic tumours (p53 labelling index = 37% and 22% respectively, P = 0.05). Other histopathological features were not related to p53 expression. In multivariate analysis, Dukes' stage (P = 0.02) and histological grade (P = 0.05) stood out as independent markers for prognosis. p53 expression was not an independent marker for prognosis. At present, p53 expression is not a useful marker for long-term prognosis. Further insight into the relationship between p53 mutations and p53 expression is needed to elucidate more precisely the clinical relevance of p53 alterations.


British Journal of Cancer (1995) 71, 1257-1262

© 1995 Stockton Press All rights reserved 0007-0920/95 $12.00

### Evaluation of p53 protein expression as a marker for long-term prognosis in colorectal carcinoma

J-WR Mulder[1], IO Baas[1], MM Polak[1], SN Goodman[2] and GJA Offerhaus[1]

[1]*Department of Pathology, Academic Medical Center, Meibergdreef 9, 1105 AZ Amsterdam Zuid-Oost, The Netherlands;* [2]*Oncology Center, Division of Biostatistics, The Johns Hopkins Medical Institutions, 550 N Broadway, Baltimore MD 21205, USA.*

**Summary** Mutation of the p53 gene is reported to be of prognostic importance in colorectal carcinomas. Immunohistochemical staining of the accumulated p53 gene product may be a simple alternative for p53 mutation analysis. Previous studies addressing the prognostic importance of p53 expression, however, yielded contradictory results. Therefore, we evaluated the importance of p53 expression as a marker for long-term prognosis in a well-characterised study population of 109 colorectal carcinomas. After antigen retrieval with target unmasking fluid (TUF), immunostaining of p53 was performed with both monoclonal antibody DO7 and polyclonal antibody CM1. Objective quantification of the p53 signal was assessed by a computerised image analyser. p53 expression was higher in non-mucinous tumours than in mucinous tumours (p53 labelling index = 30% and 17% respectively, $P = 0.05$), and in metastatic tumours compared with non-metastatic tumours (p53 labelling index = 37% and 22% respectively, $P = 0.05$). Other histopathological features were not related to p53 expression. In multivariate analysis, Dukes' stage ($P = 0.02$) and histological grade ($P = 0.05$) stood out as independent markers for prognosis. p53 expression was not an independent marker for prognosis. At present, p53 expression is not a useful marker for long-term prognosis. Further insight into the relationship between p53 mutations and p53 expression is needed to elucidate more precisely the clinical relevance of p53 alterations.

**Keywords:** p53; long-term; prognosis; colorectal; carcinoma

The p53-suppressor gene is the most frequently altered gene in solid human malignancies (reviewed in Lane, 1992; Levine, 1992*a*, 1992*b*; Oren, 1992; Vogelstein and Kinzler, 1992). It is located on the short arm of chromosome 17 in the region 17p13 and encodes a 53 kDa nuclear phosphoprotein that serves as a transcription factor (Kern *et al.*, 1992; El-Deiry *et al.*, 1993). The p53 protein indirectly regulates cell growth and inhibits cells with mutagenic damage from entering the S-phase by arresting the cell cycle in $G_1$, during which DNA repair can proceed.

In colorectal cancer, p53 mutations are frequently accompanied by allelic loss of 17p (Baker *et al.*, 1989; Rodrigues *et al.*, 1990). Both p53 mutations and allelic deletion of 17p occur late in tumour progression (Baker *et al.*, 1990) and are reported to have prognostic value after surgery (Kern *et al.*, 1989; Laurent-Puig *et al.*, 1992; Offerhaus *et al.*, 1992; Hamelin *et al.*, 1994). However, both detection of p53 mutations at the DNA level and detection of allelic deletion of 17p by restriction fragment length polymorphism (RFLP) analysis are cumbersome procedures and therefore not feasible in routine diagnosis.

Recent reports describe a strong relation between p53 gene mutations and mutant p53 protein expression (Rodrigues *et al.*, 1990; de Angelis *et al.*, 1993; Baas *et al.*, 1994). The mutant p53 protein is characterised by a conformational change resulting in prolonged half-life and stability, enabling its detection by routine immunohistochemical (IHC) techniques (Finlay *et al.*, 1988). Therefore, immunostaining of the p53 protein may be an important surrogate test for p53 mutation analysis.

In solid neoplasms including carcinomas of the breast (Barnes *et al.*, 1993), stomach (Martin *et al.*, 1992; Starzynska *et al.*, 1992), lung (Quinlan *et al.*, 1992), ovary (Bosari *et al.*, 1993) and pancreas (DiGuiseppe *et al.*, 1994), p53 expression has been correlated with shortened survival. In colorectal carcinomas, however, a correlation between survival and nuclear p53 expression has not been consistently observed (Scott *et al.*, 1991; Remvikos *et al.*, 1992; Starzynska *et al.*, 1992; Sun *et al.*, 1992; Yamaguchi *et al.*, 1992; Bell *et al.*, 1993; Bosari *et al.*, 1994; Nathanson *et al.*, 1994). The contradictory results of these studies might be partly due to the variability in IHC techniques used. Moreover, most follow-up studies lacked statistical power owing to relatively small patient populations or limited follow-up periods.

Therefore, in this study we analysed the value of p53 protein expression for long-term prognosis in a large, well-characterised study population with over 20 years of follow-up. p53 expression was evaluated by two different anti-p53 antibodies, which in a previous study stood out as being most accurate for p53 protein detection and association with p53 gene mutation (Baas *et al.*, 1994). p53 expression was objectively scored by a computerised image analyser. In addition, we evaluated the relationship between p53 expression and other histopathological parameters known to be of importance in colorectal cancer.

### Materials and methods

### *Study population and follow-up*

The original study population consisted of 155 patients with colorectal carcinoma, operated on between 1967 and 1974 in the University Hospital of Leiden. The study population had previously undergone extensive research for a large number of histopathological parameters which have a bearing on tumour biology and prognosis (Bloem, 1983; Offerhaus *et al.*, 1991). For the present p53 immunostudy, tissue blocks were available from 109 patients only. These patients did not differ significantly from the original 155 with respect to age, sex or the histopathological parameters. Histopathological parameters were determined by review of slides; location of the tumour and macroscopic aspect, together with the patient characteristics, were collected from the medical records by review of charts.

In the p53-tested cohort of 109 patients, there were 56 men and 53 women; the median age was 66 years (mean age 65 years, s.d. = 10 years, range 25–96 years). Twenty-two tumours were located in the caecum or ascending colon, eight in the transverse colon or splenic flexure, 46 in the descending colon or sigmoid and 33 tumours in the rectum. Tumours

Correspondence: GJA Offerhaus
Received 27 July 1994; revised 8 December 1994; accepted 19 December 1994

were staged according to the modified Dukes' classification (Dukes, 1932; Turnbull *et al.*, 1967). Eighteen patients had a Dukes' A carcinoma (confined within the muscularis propria), 61 patients had a Dukes' B carcinoma (extension through the muscularis propria into the pericolic fat), 27 patients had a Dukes' C carcinoma (positive regional lymph nodes without distant metastases), and three patients had a Dukes' D carcinoma (either invasion of adjacent organs or evidence of distant metastases). Twenty carcinomas were well differentiated, 67 were moderately differentiated and 22 were poorly differentiated. Twenty-nine of the 109 tumours were mucinous carcinomas (defined as at least 30% of the volume being occupied by mucine lakes) (Mecklin *et al.*, 1986). Of those cases in which the macroscopic aspect was reliably reported, 34 tumours showed exophytic growth and 55 tumours showed ulcerative growth. Fourteen tumours showed 'Crohn's-like' lymphocytic infiltration (Jass, 1986; Graham and Appelman, 1990); in five cases the presence or absence of lymphocytic infiltration was not evaluable. In 102 tumours vasoinvasion of tumour cells was studied by Van Gieson's elastic stain and a factor VIII immunoperoxidase method for the localisation of endothelial cells (Mukai *et al.*, 1980; Muller *et al.*, 1989; Offerhaus *et al.*, 1991).

Follow-up was obtained through physician contact and ended on 30 September 1993.

### *Immunohistochemistry for p53*

On 109 paraffin-embedded specimens, routine immunostaining was performed as reported previously (Baas *et al.*, 1994), using target unmasking fluid ('TUF'; Kreatech Technology, Amsterdam, The Netherlands) to enhance antigen retrieval (van den Berg *et al.*, 1993). Both rabbit polyclonal antibody CM1 (Novacastra laboratories, Newcastle upon Tyne, UK) and mouse monoclonal antibody DO7 (Dakopatts, Glostrup, Denmark) against the p53 protein served as primary antibodies (Baas *et al.*, 1994). Further staining was with the streptavidin-horseradish peroxidase (HRP)-ABC method (Vectastain, Vector Laboratories, Burlingame, CA, USA), and the chromagen was diaminobenzidine (DAB). Nuclear counterstaining was performed with methyl green, enabling p53 protein quantification by an image analyser (Baas *et al.*, 1994). Staining was controlled by omission of the primary antibody.

### *Image analysis*

The CAS 200 image analysis system consists of a conventional microscope with mounted television camera which is linked to a computer and colour monitor. The ER/PR software program enabled measurement of the total area of positive nulcear staining in any selected microscopic field, while the methyl green nuclear counterstain enabled measurement of the total nuclear area. The ratio expressed as p53 labelling index (LI) gave an objective value for the percentage of positive-staining nuclei. Baseline was set on p53-negative normal mucosa. Negative stromal elements were controlled for by computing the mean p53 LI for each slide in at least five representative fields at 400 × magnification, containing between 100 and 250 tumour nuclei (Baas *et al.*, 1994).

### *Statistical analysis*

Statistical analysis was performed with JMP software (SAS Institute, Cary, NC, USA). For survival analysis p53 expression was divided into three groups: (1) no nuclear p53 expression (LI<1%), (2) low nuclear p53 expression (LI 1-30%), and (3) high nuclear p53 expression (LI >30%). This tripartition is based on the results of previous studies by our group (Baas *et al.*, 1994). Survival analysis for the other histopathological parameters was assessed in both the p53-tested cohort and the original cohort. One patient died within 30 days of surgery, and was therefore excluded from survival analysis. Kaplan-Meier survival curves were calculated and tested for significance by an univariate log-rank statistic. These curves included only colorectal cancer-related deaths as events. Deaths from other causes were treated as censored events at time of occurrence. One patient was lost to follow-up after 11.8 years and treated as a censored event from that time. The independent prognostic value of parameters was tested using the multivariate Cox regression model. Correlation between p53 expression and histopathological parameters was tested using a *t*-test statistic or analysis of variance (ANOVA) for multiple means.

### Results

### *p53 expression*

p53 immunostaining was initially evaluated by conventional light microscopy by two authors who were blinded for other

**Table I** p53 expression by immunostaining with MAb DO7 and PAb CM1

| Percentage positive cells | Estimation by conventional light microscopy DO7 n | % | CM1 n | % | Quantification by computerised image analysis Labelling index (%) | | DO7 n | % |
|---|---|---|---|---|---|---|---|---|
| <1 | 23 | 21 | 35 | 32 | LI | <1 | 31 | 28 |
| 1-30 | 17 | 16 | 25 | 23 | LI | 1-30 | 35 | 32 |
| >30 | 69 | 63 | 49 | 45 | LI | >30 | 43 | 40 |
| | 109 | 100 | 109 | 100 | | | 109 | 100 |

p53 protein positivity is evaluated by conventional light microscopy for MAb DO7 and PAb CM1. p53 labelling index (LI) is assessed on a computerised image analyser for p53 staining with MAb DO7.

**Table II** Association between IHC p53 expression with MAb DO7 and different histopathological parameters (mean p53 LI = the average of the labelling indices in the given subgroup)

| | n | Mean p53-LI (%) | P |
|---|---|---|---|
| *Sex* | | | |
| Male | 56 | 27 | |
| Female | 53 | 26 | >0.2 |
| *Age (years)* | | | |
| <66 | 54 | 25 | |
| >66 | 55 | 28 | >0.2 |
| *Location* | | | |
| Caecum ascending colon | 22 | 21 | |
| Transverse colon splenic flexure | 8 | 28 | |
| Descending colon sigmoid | 46 | 29 | |
| Rectum | 33 | 26 | >0.2 |
| *Dukes' stage* | | | |
| A | 18 | 26 | |
| B | 61 | 21 | |
| C D | 30 | 37 | 0.05 |
| *Differentiation grade* | | | |
| Good | 20 | 23 | |
| Moderate | 67 | 27 | |
| Poor | 22 | 27 | >0.2 |
| *Mucus content* | | | |
| Mucinous | 29 | 17 | |
| Non-mucinous | 80 | 30 | 0.05 |
| *Macroscopic aspect* | | | |
| Exophytic | 34 | 30 | |
| Ulcerative | 55 | 27 | >0.2 |
| *Lymphocytic infiltration* | | | |
| Absent | 90 | 27 | |
| Present | 14 | 27 | >0.2 |
| *Vasoinvasion* | | | |
| Absent | 56 | 29 | |
| 1 vessel | 25 | 18 | |
| >1 vessel | 21 | 34 | 0.13 |

variables. p53 positivity was restricted to the nuclei of the cells of malignant glands. Normal colonic mucosa expressed no p53 protein. The results of the p53 immunostaining are listed in Table I. A high correlation was found between the results of the p53 detection with MAb DO7 and pAb CM1 ($P<0.0005$). Therefore, objective quantification of p53 expression with a CAS 200 image analyser was performed only on the DO7-stained specimens.

Table I shows that the results of conventional evaluation of p53 expression slightly differ from the amount of p53 protein when quantified by a computerised image analyser. Thirty-one (28%) carcinomas showed no p53 expression (LI<1%), 35 (32%) carcinomas showed low p53 expression (LI 1–30%) and 43 (40%) carcinomas showed high p53 expression (LI>30%).

### *p53 expression and histopathological parameters*

The associations between p53 expression and histopathological parameters are listed in Table II. A weak significant overall increase in p53 expression with advancing Dukes' stage was observed ($P = 0.05$). Mucinous tumours expressed significantly less p53 protein than non-mucinous tumours ($P = 0.05$). No significant difference in p53 expression with regard to sex, age, macroscopic aspect, lymphocytic infiltration or vasoinvasion by tumour cells was observed. Results did not change significantly when low p53 expression (LI 1–30%) was regarded as negative, or when a subdivision into p53-negative (LI<1%) *vs* p53-positive (LI>1%) carcinomas was used.

### *Survival analysis*

The results of the univariate survival analysis in the p53-tested study group and the original complete cohort are listed in Table III. The median period between surgery and death or last physician contact was 7.3 years in both groups. During follow-up, in the complete cohort 57 (37%) patients died of colorectal carcinoma and 65 (42%) patients died of causes unrelated to colorectal cancer. In the p53-tested

**Table III** Prognostic importance of histopathological parameters tested by univariate log-rank analysis and the multivariate Cox regression model. The risk ratios at each level use as a reference the previous level, not the baseline level (RR = relative risk)

| | Complete cohort | | | p53-tested cohort | | |
|---|---|---|---|---|---|---|
| | n | 10 year surv (%) | P | n | 10 year surv (%) | P |
| *Univariate analysis* | | | | | | |
| Sex | | | | | | |
| Male | 81 | 56 | | 55 | 55 | |
| Female | 73 | 62 | >0.2 | 53 | 59 | >0.2 |
| Age | | | | | | |
| <median | 77 | 60 | | 54 | 58 | >0.2 |
| >median | 77 | 58 | >0.2 | 54 | 56 | |
| Location | | | | | | |
| Caecum/ascending colon | 31 | 59 | | 22 | 58 | |
| Transverse colon/splenic flexure | 12 | 57 | | 8 | 63 | |
| Descending colon/sigmoid | 64 | 62 | | 45 | 53 | |
| Rectum | 47 | 54 | >0.2 | 33 | 58 | >0.2 |
| Dukes' stage | | | | | | |
| A | 28 | 82 | | 18 | 73 | |
| B | 90 | 61 | | 61 | 60 | |
| C/D | 36 | 37 | <0.001 | 29 | 41 | 0.03 |
| Differentiation grade | | | | | | |
| Good | 27 | 76 | | 20 | 74 | |
| Moderate | 95 | 60 | | 66 | 57 | |
| Poor | 32 | 41 | 0.005 | 22 | 40 | 0.04 |
| Mucus content | | | | | | |
| Mucinous | 42 | 64 | | 29 | 73 | |
| Non-mucinous | 112 | 57 | >0.2 | 79 | 51 | 0.2 |
| Macroscopic aspect | | | | | | |
| Exophytic | 47 | 73 | | 34 | 71 | |
| Ulcerative | 77 | 54 | 0.04 | 55 | 51 | 0.13 |
| Lymphocytic infiltration | | | | | | |
| Absent | 120 | 54 | | 90 | 54 | |
| Present | 26 | 75 | 0.04 | 14 | 63 | >0.2 |
| Vasoinvasion | | | | | | |
| Absent | 74 | 60 | | 56 | 64 | |
| 1 vessel | 34 | 57 | | 25 | 67 | |
| >1 vessel | 25 | 35 | 0.03 | 21 | 38 | 0.08 |
| p53 expression (%) | | | | | | |
| LI <1 | | | | 31 | 45 | |
| LI 1–30 | | | | 35 | 74 | |
| LI >30 | | | | 43 | 52 | 0.08 |
| *Multivariate analysis* | n | *RR* | P | n | *RR* | P |
| Dukes' stage | | | | | | |
| A | 28 | 1.0 | | 18 | 1.0 | |
| B | 90 | 2.3 | | 61 | 1.5 | |
| C/D | 36 | 2.1 | <0.001 | 29 | 1.9 | 0.02 |
| *Differentiation grade* | | | | | | |
| Well | 27 | 1.0 | | 20 | 1.0 | |
| Moderate | 95 | 1.6 | | 66 | 1.5 | |
| Poorly | 32 | 1.9 | 0.01 | 22 | 1.9 | 0.05 |

cohort these numbers were 42 (39%) and 48 (44%) respectively.

In the complete cohort, increase in Dukes' stage (Figure 1a), poorer grade of differentiation, ulcerative growth, lymphocytic infiltration and vasoinvasion was related to worse prognosis. In the smaller p53 study subset, only Dukes' stage (Figure 1b) and grade of differentiation remained significant. The amount of p53 expression in carcinomas showed a tendency towards an association with patient survival ($P = 0.08$), but the pattern of this relationship is difficult to interpret: the highest and lowest p53 categories showed the poorest survival, whereas the intermediate p53 category showed the best prognosis (Figure 1c).

In the complete cohort, multivariate analysis showed that only Dukes' stage ($P = <0.001$) and differentiation grade ($P = 0.01$) were independent markers for prognosis. These parameters were also independent predictors of prognosis in the smaller p53-tested cohort (Dukes' stage, $P = 0.02$; differentiation grade, $P = 0.05$) (Table III).

### Discussion

The prognosis after seemingly curative resection of colorectal carcinoma depends largely on the absence or presence of occult metastases, often accounting for mortality. Prediction of outcome is currently based mainly on the stage of colorectal carcinoma at time of resection. However, patients with tumours of the same stage often show dramatically different outcome. Therefore, more specific prognostic markers would provide a rationale to adjust different therapeutic approaches. Alterations of the p53 tumour-suppressor gene are potentially such a marker (Hamelin *et al.*, 1994), and immunostaining of the p53 protein product could be a valuable test for p53 gene alterations (Rodrigues *et al.*, 1990; de Angelis *et al.*, 1993; Baas *et al.*, 1994).

Previous studies addressing the prognostic value of p53 were mostly restricted to short-term follow-up, and in particular the IHC studies yielded variable results (Table IV). This variability might come from several causes. First of all, these study groups may not always be comparable. Moreover, the use of various antibodies against different epitopes of the p53 protein, and sometimes the use of antigen retrieval systems, may also account for some of the variability in the percentages of p53 positivity seen among the different studies (Table IV) (van den Berg *et al.*, 1993; reviewed in Wynford-Thomas, 1992; Hall and Lane, 1994). We previously evaluated various procedures and six different p53 antibodies in relationship with underlying p53 changes and selected the two most accurate procedures for p53 protein detection for use in this study (Baas *et al.*, 1994). Objective quantification of p53 expression was achieved by use of a computerised image analyser. Optimised IHC techniques combined with computerised quantification yielded p53 positivity in 72% of the carcinomas (Table I), a percentage that exceeds that of all previous studies (Table IV).

In our study, p53 expression was higher in metastatic carcinomas (LI = 37%) than in non-metastatic Dukes' B carcinomas (LI = 21%). However, this phenomenon is not consistently observed in other studies (Campo *et al.*, 1991; Scott *et al.*, 1992; Purdie *et al.*, 1991; Remvikos *et al.*, 1992; Starzynska *et al.*, 1992; Sun *et al.*, 1992; Bell *et al.*, 1993; de Angelis *et al.*, 1993; Bosari *et al.*, 1994; Mulder *et al.*, 1995). As in other studies (Campo *et al.*, 1991; Hanski *et al.*, 1992), mucinous carcinomas exhibited significantly less p53 protein than non-mucinous carcinomas, suggesting more p53 mutations in non-mucinous carcinomas. Together with other molecular aspects (Kern *et al.*, 1989; Laurent-Puig *et al.*, 1991) and similar findings in the ovary (Enomoto *et al.*, 1991), this finding suggests that mucinous tumours may be biologically different. No correlation was found between p53 expression and the other histopathological parameters. In this study, we did not observe a difference in p53 positivity between right- and left-sided carcinomas. This contrasts with some studies in which p53-positive tumours were predominantly found in the distal part of the large bowel (Scott *et al.*, 1991; Remvikos *et al.*, 1992; Starzynska *et al.*, 1992; Bosari *et al.*, 1994), but is concordant with other studies (Purdie *et al.*, 1991; Hanski *et al.*, 1992; Yamaguchi *et al.*, 1992; Bell *et al.*, 1993; Nathanson *et al.*, 1994). The other results fit in the general picture derived from other studies (Campo *et al.*,

**Figure 1** Kaplan–Meier survival curves according to Dukes' classification in the original complete cohort (**a**) and the smaller p53-tested study group (**b**), and according to p53 expression in p53-tested group (**c**). Prognostic importance was tested by univariate log-rank statistic. Subjects at risk are indicated in the figures. Increase in Dukes' stage was significantly related to survival in both the complete cohort and the p53-tested group ($P<0.001$ and $P = 0.03$ respectively). p53 expression showed a tendency towards a relationship with survival ($P = 0.08$), but the pattern of the relationship is difficult to interpret: the highest and lowest p53 categories showed the poorest survival, whereas the intermediate p53 category showed the best prognosis.

**Table IV** Survey of recent studies addressing p53 expression in colorectal carcinomas (crc) by immunostaining. When more than one antibody was evaluated, the antibody (Ab) yielding the highest staining percentage is listed. When studied, the importance for prognosis is listed

| Reference | Per cent of crc expressing p53 (Ab) | Antigen enhancement | Other antibodies evaluated | n | Follow-up in years Mean range | Prognostic value Univariate P | Multivariate P |
|---|---|---|---|---|---|---|---|
| This study | 72 (DO7) | TUF[a] | CM1 | 109 | 7.3[b] 0-28 | NS | NS |
| Starzynska et al. (1992) | 46 (CM1) | — | — | 107 | <1 0-1 | <0.001 | — |
| Bell et al. (1993) | 45 (421) | — | 240/1801 | 100 | 3 0-8 | NS | — |
| Yamaguchi et al. (1992) | 61 (1801) | — | — | 100 | 3 0.5-4 | 0.01 | <0.05[c] |
| Scott et al. (1991) | 42 (421) | — | — | 52 | 3 1-7 | NS | — |
| Remvikos et al. (1992) | 60 (240) | — | 421/1801 | 78 | 3.5[b] 0-4 | <0.05 | — |
| Bosari et al. (1993) | 46 (1801) | Saponin | CM1 | 206 | >5 5-9 | <0.02 | NS |
| Sun et al. (1992) | 24 (CM1) | — | 1801 | 293 | ? 1-8 | NS | NS |
| Nathanson et al. (1994) | 62 (1801) | ARS[d] | — | 84 | ? 5-10 | NS | NS |

[a]Target unmasking fluid. [b]Median. [c]No Dukes' stage included in multivariate analysis. [d]Antigen retrieval system.

1991; Purdie *et al.*, 1991; Scott *et al.*, 1991; Hanski *et al.*, 1992; Remvikos *et al.*, 1992; Starzynska *et al.*, 1992; Yamaguchi *et al.*, 1992; Bell *et al.*, 1993; de Angelis *et al.*, 1993; Kaklamanis *et al.*, 1993; Bosari *et al.*, 1994; Nathanson *et al.*, 1994).

Established markers for prognosis such as Dukes' stage and differentiation grade were independently associated with survival, validating this study population. In this long-term follow-up study we found that nuclear p53 protein expression is not related to outcome after surgery. This result is similar to previous studies (Scott *et al.*, 1991; Sun *et al.*, 1992; Bell *et al.*, 1993; Nathanson *et al.*, 1994). Yamaguchi *et al.* (1992) reported p53 positivity to be of independent prognostic importance, but in their multivariate analysis Dukes' stage was not included. Both Bosari *et al.* (1994) and Sun *et al.* (1992) found cytoplasmic p53 staining with PAb CM1 to be of independent prognostic importance, but as in most other studies no reliable cytoplasmic staining was found in our study.

The lack of consistent prognostic value of nuclear p53 protein expression might indicate that the reported importance for prognosis of p53 mutation needs additional study and/or that the relationship between immunostaining of p53 protein and p53 gene mutation might be too much confounded by other biological mechanisms and technical caveats (reviewed in Wynford-Thomas, 1992; Hall and Lane, 1994).

In conclusion, this study indicates that, with current methodology, p53 protein expression does not appear to contribute to the prediction of long-term prognosis after resection of colorectal carcinoma. We emphasise that further insight into the relationship between p53 gene mutations and p53 protein expression is needed to elucidate more precisely their clinical relevance.

**Acknowledgements**
JWR Mulder carried out this study as a visiting scientist at the Department of Pathology, The Johns Hopkins Medical Institutions, Baltimore, USA, with financial support from the Netherlands Digestive Diseases Foundation. We are indebted to our colleagues of the Department of Pathology, University Hospital Leiden, The Netherlands, for the availability of the tissue samples. We thank Dr Stanley R Hamilton, Department of Pathology, The Johns Hopkins Medical Institutions, Baltimore, USA, for reviewing the manuscript,

### References

DE ANGELIS P, STOKKE T, SMEDSHAMMER L, LOTHE RA, MELING GI, ROFSTAD M, CHEN Y AND CLAUSEN OPF. (1993). p53 expression is associated with a high degree of tumor DNA aneuploidy and incidence of p53 gene mutation, and is localized to the aneuploid component in colorectal carcinomas. *Int. J. Oncol.*, **3**, 305-312.

BAAS IO, MULDER JWR, OFFERHAUS GJA, VOGELSTEIN B AND HAMILTON SR. (1994). An evaluation of six antibodies for immunohistochemistry of mutant p53 gene product in archival colorectal neoplasms. *J. Pathol.*, **172**, 5-12.

BAKER SJ, FEARON ER, NIGRO JM, HAMILTON SR, PREISINGER AC, JESSUP JM, VAN TUINEN P, LEDBETTER DH, BARKER DF, NAKAMURA Y, WHITE R AND VOGELSTEIN B. (1989). Chromosome 17p deletions and p53 gene mutations in colorectal carcinomas. *Science*, **244**, 217-221.

BAKER SJ, PREISINGER AC, JESSUP M, PARASKEVA C, MARKOWITZS S, WILLSON JKV, HAMILTON S AND VOGELSTEIN B. (1990). p53 gene mutations occur in combination with 17p allelic deletions as late events in colorectal tumorigenesis. *Cancer Res.*, **50**, 7717-7722.

BARNES DM, DUBLIN EA, FISHER CJ, LEVISON DA AND MILLIS RR. (1993). Immunohistological detection of p53 in mammary carcinoma: an important new independent indicator of prognosis? *Hum. Pathol.*, **24**, 469-476.

BELL SM, SCOTT N, CROSS D, SAGAR P, LEWIS FA, BLAIR GE, TAYLOR GR, DIXON MF AND QUIRKE P. (1993). Prognostic value of p53 overexpression and c-Ki-ras gene mutations in colorectal cancer. *Gastroenterology*, **104**, 57-64.

VAN DEN BERG FM, BAAS IO, POLAK MM AND OFFERHAUS GJA. (1993). Detection of p53 overexpression in routinely paraffin-embedded tissue of human carcinomas using a novel target unmasking fluid. *Am. J. Pathol.*, **142**, 381-385.

BLOEM R. (1983). Colorectal carcinoma. Dissertation, University of Leiden, The Netherlands.

BOSARI S, VIALE G, RADAELLI U, BOSSI P, BONOLDI E AND COGGI G. (1993). p53 accumulation in ovarian carcinomas and its prognostic implications. *Hum. Pathol.*, **24**, 1175-1179.

BOSARI S, VIALE G, BOSSI P, MAGGIONI M, COGGI G, MURRAY JJ AND LEE AKC. (1994). Cytoplasmatic accumulation of p53 protein: an independent prognostic indicator in colorectal adenocarcinomas. *J. Natl Cancer. Inst.*, **86**, 681-687.

CAMPO E, DE LA CALLE-MARTIN O, MIQUEL R, PALACIN A, ROMERO M, FABREGAT V, VIVES J, CARDESA A AND YAGUE J. (1991). Loss of heterozygosity of p53 gene and p53 protein expression in human colorectal carcinomas. *Cancer Res.*, **51**, 4436-4442.

DIGUISEPPE JA, HRUBAN RH, GOODMAN SN, ALLISON DA, CAMERON JL AND OFFERHAUS GJA. (1994). Overexpression of the p53 tumor suppressor protein in pancreatic adenocarcinoma. *Am. J. Clin. Pathol.*, **101**, 684-688.

DUKES CE. (1932). The classification of cancer of the rectum. *J. Pathol. Bacteriol.*, **35**, 323-332.

EL-DEIRY WS, TOKINO T, VELCULESCU VE, LEVY DB, PARSONS R, TRENT JM, LIN D, MERCER WE, KINZLER KW AND VOGELSTEIN B. (1993). WAF1, a potential mediator of p53 tumor suppression. *Cell*, **75**, 817-825.

ENOMOTO T, WEGHORST CM, INOUE M, TANIZAWA O AND RICE JM. (1991). K-ras activation occurs frequently in mucinous adenocarcinomas and rarely in other common epithelial tumors of the human ovary. *Am. J. Pathol.*, **139**, 777-785.

FINLAY CA, HINDS PW, TAN TH, ELIYAHU D, OREN M AND LEVINE AJ. (1988). Activating mutations for transformation by p53 produce a gene product that forms an hsc700-p53 complex with an altered half-life. *Mol. Cell. Biol.*, **8**, 531-539.

GRAHAM DM AND APPELMAN HD. (1990). Crohn's-like lymphoid reaction and colorectal carcinomas: a potential histologic prognosticator. *Modern Pathol.*, **3**, 332-335.

HALL PA AND LANE DP. (1994). p53 in tumour pathology: can we trust immunohistochemistry? revisited! *J. Pathol.*, **172**, 1-4.

HAMELIN R. LAURENT-PUIG P. OLSCHWANG S. JEGO N. ASSELAIN B. REMVIKOS Y. GIRODET J. SALMON RJ AND THOMAS G. (1994). Association of p53 mutations with short survival in colorectal cancer. *Gastroenterology*, **106**, 42-48.

HANSKI C. BORNHOEFT G. SHIMODA T. HANSKI M-L. LANE DP. STEIN H AND RIECKEN E-O. (1992). Expression of p53 protein in invasive colorectal carcinomas of different histologic types. *Cancer*, **70**, 2772-2777.

JASS JR. (1986). Lymphocytic infiltration and survival in rectal cancer. *J. Clin. Pathol.*, **39**, 585-589.

KAKLAMANIS L. GATTER KC. MORTENSEN N. BAIGRIE RJ. HERYET A. LANE DP AND HARRIS AL. (1993). p53 expression in colorectal adenomas. *Am. J. Pathol.*, **142**, 87-93.

KERN SE. FEARON ER. TERSMETTE KWF. ENTERLINE JP. LEPPERT M. NAKAMURA Y. WHITE R. VOGELSTEIN B AND HAMILTON SR. (1989). Clinical and pathological associations with allelic loss in colorectal carcinoma. *J. Am. Med. Assoc.*, **261**, 3099-3103.

KERN SE. PIETENPOL JA. THIAGALINGAM S. SEYMOUR. A. KINZLER KW AND VOGELSTEIN B. (1992). Oncogenic forms of p53 inhibit p53-regulated gene expression. *Science*, **256**, 827-830.

LANE DP. (1992). p53, guardian of the genome. *Nature*, **358**, 15-16.

LAURENT-PUIG P. OLSCHWANG S. DELATTRE O. VALIDIRE P. MELOT T. MOSSERI V. SALMON RJ AND THOMAS G. (1991). Association of Ki-ras mutation with differentiation and tumor-formation pathways in colorectal carcinoma. *Int. J. Cancer*, **49**, 220-223.

LAURENT-PUIG P. OLSCHWANG S. DELATTRE O. REMVIKOS V. ASSELAIN B. MELOT T. VALIDIRE P. MULERIS M. GIRODET J. SALMON RJ AND THOMAS G. (1992). Survival and acquired genetic alterations in colorectal cancer. *Gastroenterology*, **102**, 1136-1141.

LEVINE AJ. (1992*a*). The p53 tumor-suppressor gene. *N. Engl. J. Med.*, **326**, 1350-1351.

LEVINE AJ. (1992*b*). The p53 tumor suppressor gene and product. *Cancer Surv.*, **12**, 59-80.

MARTIN HM. FILIPLE MI. MORRIS RW. LANE DP AND SILVESTRE F. (1992). p53 expression and prognosis in gastric carcinoma. *Int. J. Cancer*, **50**, 859-862.

MECKLIN JP. SIPPONEN P AND JÄRVINEN HJ. (1986). Histopathology of colorectal carcinomas and adenomas in cancer family syndrome. *Dis. Colon Rectum*, **29**, 849-853.

MUKAI K. ROSAI J AND BURGDORF WHC. (1980). Localization of factor VIII-related antigen in vascular endothelial cells using an immunoperoxidase method. *Am. J. Surg. Pathol.*, **4**, 273-276.

MULDER JWR. WIELENGA VJM. POLAK MM. VAN DEN BERG FM. ADOLF GR. HERRLICH P. PALS ST AND OFFERHAUS GJA. (1995). Expression of mutant p53 protein and CD44 variant proteins in colorectal tumorigenesis. *Gut*, **36**, 76-80.

MULLER S. CHESNER IM. EGAN MJ. ROWLANDS DC. COLLARD MJ. SWARBRICK ET AND NEWMAN J. (1989). Significance of venous and lymphatic invasion in malignant polyps of the colon and rectum. *Gut*, **30**, 1385-1391.

NATHANSON SD. LINDEN MD. TENDER P. ZARBO RJ. JACOBSON G AND NELSON LS. (1994). Relationship among p53, stage, and prognosis of large bowel cancer. *Dis. Colon Rectum*, **37**, 527-534.

OFFERHAUS GJA. GIARDIELLO FM. BRUIJN JA. STIJNEN T. MOLYVAS EN AND FLEUREN GJ. (1991). The value of collagen IV immunohistochemistry in colorectal cancer. *Cancer*, **67**, 99-105.

OFFERHAUS GJA. DE FEYTER EP. CORNELISSE CJ. TERSMETTE KWF. FLOYD J. KERN SE. VOGELSTEIN B AND HAMILTON SR. (1992). The relationship of DNA aneuploidy to molecular genetic alterations in colorectal carcinoma. *Gastroenterology*, **102**, 1612-1619.

OREN M. (1992). p53 - the ultimate tumor suppressor gene. *FASEB. J.*, **6**, 3169-3176.

PURDIE CA. O'GRADY JO. PIRIS J. WYLLIE AH AND BIRD CC. (1991). p53 expression in colorectal tumors. *Am. J. Pathol.*, **138**, 807-813.

QUINLAN DC. DAVIDSON AG. SUMMERS CL. WARDEN HE AND DOSHI HM. (1992). Accumulation of p53 protein correlates with a poor prognosis in human lung cancer. *Cancer Res.*, **52**, 4828-4831.

REMVIKOS Y. TOMINAGA O. HAMMEL P. LAURENT-PUIG P. SALMON RJ. DUTRILLAUX B AND THOMAS G. (1992). Increased p53 protein content of colorectal tumours correlates with poor survival. *Br. J. Cancer*, **66**, 758-764.

RODRIGUES NR. ROWAN A. SMITH ME. KERR IB. BODMER WF. GANNON JV AND LANE DP. (1990). p53 mutations in colorectal cancer. *Proc. Natl Acad. Sci. USA*, **87**, 7555-7559.

SCOTT NP. SAGAR P. STEWART J. BLAIR GE. DIXON MF AND QUIRKE P. (1991). p53 in colorectal cancer: clinicopathological correlation and prognostic significance. *Br. J. Cancer*, **63**, 317-319.

STARZYNSKA T. BROMLEY M. GHOSH A AND STERN PL. (1992). Prognostic significance of p53 overexpression in gastric and colorectal carcinoma. *Br. J. Cancer*, **66**, 558-562.

SUN X-F. CARSTENSEN JM. ZHANG H. STÅL O. WINGREN S. HATSCHEK T AND NORDENSKJÖLD B. (1992). Prognostic significance of cytoplasmic p53 oncoprotein in colorectal adenocarcinoma. *Lancet*, **340**, 1369-1373.

TURNBULL JR RB. KYLE K. WATSON FR AND SPRATT J. (1967). Cancer of the colon; the influence of the *no-touch isolation* technic on survival rates. *Ann. Surg.*, **166**, 420-427.

VOGELSTEIN B AND KINZLER KW. (1992). p53 function and dysfunction. *Cell*, **70**, 523-526.

WYNFORD-THOMAS D. (1992). p53 in tumour pathology: can we trust immunohistochemistry? *J. Pathol.*, **166**, 329-330.

YAMAGUCHI A. KUROSAKA Y. FUSHIDA S. KANNO M. YONEMURA Y. MIWA K AND MIYAZAKI I. (1992). Expression of p53 protein in colorectal cancer and its relationship to short-term prognosis. *Cancer*, **70**, 2778-2784.